# The Holt Ockley System of Nursing

**Published:** 1909-08-28

**Authors:** 


### Nursing Administration.

### THE HOLT OCKLEY SYSTEM OF NURSING.

### I.—NURSES AND PATIENTS.

The recently published report of the proceedings at the Jubilee Congress of Nursing recalls to mind the only controversial note which arose in the course of the discussions. This discussion arose in regard to the system practised by the Cottage Benefit Association, commonly known as the Holt Ockley system. It may be desirable, in the interests of district nursing, to examine this system a little more closely than was possible during the Liverpool meetings, with a view to forming an opinion on its special features.

The Cottage Benefit Association was founded twenty-seven years ago, and has now 137 branches working on the Holt Ockley system, employing among them 500 nurses. The peculiar features which distinguish these nurses from ordinary village nurses are two in number—very short training, and residence in the cottage of the patient. It is claimed for these two points in the organisation that they make for economy and efficiency; in other words, that they are arranged in the interests of nurses, patients, and subscribers. Branches desirous of securing nurses are invited to send village women to undergo a four months' or a six months' course, at a cost of from £15 to £20, which can, if desired, be paid in yearly instalments.

The nurses are not encouraged to go on to take a course of midwifery, although, in response to the demand for midwives about eight per cent. of the Holt Ockley nurses have now qualified themselves in this respect. The promoters desire it to be understood that the women who receive this preparation for their duties are not qualifying for "assisting at surgical operations, or such surgical cases as could not properly be treated in a labourer's cottage," but this is a distinction easier to enforce on paper than in emergency. The conditions on which the nurses are supplied differ in essential particulars to those usually governing district work. In place of the nurses working from their own quarters and visiting several patients a day, under the Holt Ockley system they reside in the cottages of the sick, and can therefore undertake only one case at a time. They are instructed that their duties consist not merely in attendance in the sick-room, but in taking the place of the mother, should she be the patient, or in assisting her in general household duties should the husband or one of the children be the invalid. She is to cook for the family, clean the cottage, dress and wash the children, and, in fact, act as a charwoman during the time when she is not engaged in the sick-room. It is this part of the system by which the promoters of the Cottage Benefit Association must stand or fall. We have watched many nursing associations begin their career with a minimum qualification for the nurses, and gradually extend the term of training until they were brought into line with the soundest nursing opinion in the country.

But the important point to consider is whether the resident nurse, prepared to act as charwoman, is a greater benefit in the cottage than the visiting district nurse. This matter is to some extent bound up with the short period of training, for undoubtedly the more thoroughly a nurse is trained the greater the waste involved in employing her outside the sphere of her patient's room. Enquiries into the system lead us to believe that the principle of supplying in one person a nurse for the patient and a charwoman for the cottage is essentially unsound; unfair to the nurses, however imperfectly equipped they may be for their nursing duties, and both unnecessary and inconvenient to the patients. For the nurses the inconveniences lie on the surface. The accommodation available for them is always poor, and sometimes very bad indeed. No cottages in rural England are intended for the accommodation of a resident nurse, and the privacy, cleanliness, and quiet to which the nurse is entitled when off duty are impossible of attainment in even the better class of cottage. The hardships endured by the Holt Ockley nurses in the worse kind of cottage are better imagined than described. No nurse who is worth her salt ever grumbles at the task of reducing her patient's surroundings to order, however bad she may find them; but she is entitled to her bath and to a peaceful night's repose in pure air after the day is done, luxuries practically unattainable in the ordinary cottage. Again, the rule that the patient provides the nurse's food during the duration of the case presses very hardly on the nurse. Often the food which is procurable is quite unsuited to the nurse's requirements; often the nurse shrinks from adding to the expenses of her patient and satisfies herself on potatoes and bread. Gastric troubles are in consequence not infrequent, though the hardships endured by the nurses seldom come to light, and can only be guessed at from the fact that they seldom continue this line of work after the completion of their contract. It is doubtful whether, from the patient's point of view, the resident cottage nurse is an unmixed boon. Not every woman while upstairs is pleased at the thought of a stranger assuming her duties, taking meals with her husband, and doing everything a little better than she can do it herself. The process of child-bearing is associated in all classes of women with causeless outbreaks of jealousy, and there are certainly inconveniences to balance the admittedly good services rendered by the nurses. It is highly creditable both to these women, with their short period of training, and to those responsible for teaching them, that complaints are infrequent. The system may be said to work at its best in districts where the patients are principally drawn from the class of gentlemen's servants, and the cottages are of the comfortable order.

